# Genetic Variants of *SLC22A1* rs628031 and rs622342 and Glycemic Control in T2DM Patients from Northern Mexico

**DOI:** 10.3390/genes16020139

**Published:** 2025-01-24

**Authors:** Janette G. Moreno-González, Sandra A. Reza-López, Everardo González-Rodríguez, Tania Samanta Siqueiros-Cendón, Alfonso Escareño Contreras, Quintín Rascón-Cruz, Irene Leal-Berumen

**Affiliations:** 1 Facultad de Ciencias Químicas, Universidad Autónoma de Chihuahua, Campus II, Circuito Universitario S/N, Chihuahua CP 31125, Mexico; jgmoreno@uach.mx (J.G.M.-G.); tsiqueiros@uach.mx (T.S.S.-C.); 2 Facultad de Medicina y Ciencias Biomédicas, Universidad Autónoma de Chihuahua, Campus II, Circuito Universitario S/N, Chihuahua CP 31109, Mexico; sreza@uach.mx (S.A.R.-L.); egonzalez@uach.mx (E.G.-R.); 3 Instituto Municipal de Pensiones, Calle Rio Sena #1100, Colonia Alfredo Chávez, Chihuahua CP 31410, Mexico; alfonso.escareno@mpiochih.gob.mx

**Keywords:** metformin, T2DM, gene variant, polymorphism, single nucleotide polymorphism (SNP), *SLC22A1*, *OCT1*

## Abstract

Background: Type 2 diabetes mellitus (T2DM) and its associated complications are of public health concern. Metformin is the most common pharmacological T2DM treatment, distributed through organic cation transporters (*OCT*s). The solute transporter family 22A1 (*SLC22A1*) gene encodes *OCT1*, and its variants may play a role in glycemic control. This study analyzed seven *SLC22A1* gene variants and their potential association with glycemic control in patients from Northern Mexico with T2DM undergoing metformin monotherapy. Methods: This cross-sectional study included 110 patients. We analyzed HbA1c values as a continuous variable and according to glycemic control categories (<7% vs. ≥7%). DNA from blood samples was genotyped using genotyping assays based on real-time PCR and PCR-RFLP. Results: Patients with GG or AA rs628031 genotypes were 2.7 times more likely to have inadequate glycemic control than those with the GA genotype (*p* = 0.042). We analyzed the combination of rs628031 and rs622342 as diplotypes. The relation between HbA1c and these diplotypes was influenced by BMI and the metformin dose. Carriers of at least one minor allele of A-rs628031 and C-rs622342 had lower HbA1c values than individuals homozygous for the major allele in both genes. Conclusions: The rs628031 and rs622342 variants are associated with lower HbA1c levels in T2DM patients. Larger studies are needed to confirm these associations.

## 1. Introduction

Type 2 diabetes mellitus (T2DM) represents around 90% of all diabetes types and it is a worldwide public health concern [1]. The incidence of complications such as cardiovascular disease, neuropathy, nephropathy, and retinopathy can be reduced through optimal hyperglycemia management [2]. However, the prevalence of glycemic control (<7.0% HbA1c) varies widely, ranging from 7% to 54% [3]. An integral T2DM treatment involves lifestyle modifications such as diet, exercise, weight management, and pharmacological interventions [2].

Metformin, a biguanide and the most widely prescribed drug for T2DM, primarily acts by the inhibition of the complex I mitochondrial respiratory chain, activating AMP-activated protein kinase (AMPK), suppressing hepatic gluconeogenesis and improving insulin sensitivity, thereby reducing blood glucose levels [4]. It is the first-line therapy following diagnosis and is also used to prevent or delay the onset of diabetes in individuals with prediabetes or those at high risk of developing type 2 diabetes [5]. In Mexico, 23-49% of T2DM patients treated with metformin do not achieve glycemic control [6,7,8].

Metformin cell distribution is achieved through organic cation transporters (*OCT*s) such as the solute transporter family 22A1 (*SLC22A1*). The three *OCT* genes, -*OCT1*, *OCT2,* and *OCT3*-, are all encoded on chromosome 6q25.3 [9,10]. The *OCT1* Online Mendelian Inheritance in Man database number is OMIN 602607; it is expressed mainly in the liver and other tissues such as skeletal muscle, small intestine, lung, and kidney, and it plays a role in the metformin response [4,11,12]. Over 80 single nucleotide polymorphisms (SNPs) have been identified in the *SLC22A1* gene [13]; some of them have been reported to modify *OCT1* transport function. A large meta-analysis in T2DM patients with European ancestry identified three polymorphisms (rs622342, rs72552763, and rs12208357) that play a role in metformin pharmacokinetics [14]. Differences in glycemic control in patients with T2DM treated with metformin suggest that there is variability in metformin responses among different ethnic groups [15,16].

One of the most studied variations in the *SLC22A1* gene related to the metformin response is rs628031 (1222A>G), which is associated with a methionine to valine substitution at codon 408 (M408V) [15,17]. Studies in Arabian (combined therapy) and Chinese Han (metformin monotherapy) found that individuals with GG or GA genotypes exhibit greater levels of HbA1c, suggesting a low response to metformin [18,19]. Conversely, in a Mexican population (metformin monotherapy), the AA genotype showed increased HbA1c values [8]. Other studies in Iranian with metformin monotherapy, or Caucasian and Mexican populations with combined therapy (metformin + sulfonylureas or metformin + sulfonylureas + insulin, respectively), did not find a significant association between this SNP and the metformin response [20,21,22].

rs622342 (1386A>C), located in an intronic region between exons 8 and 9, does not cause an amino acid change [22,23]. This variant has been linked to a positive response to metformin. For instance, in the South Indian population, individuals carrying the AA genotype showed a 5.6 times better response to metformin compared to those with the CC genotype after 12 weeks on metformin monotherapy [23]. Similar studies conducted in Caucasian and Mexican populations found an association between the A-rs622342 allele and lower HbA1c levels [8,22]. A study in a Jordanian population did not show a relationship between this variant and adequate glycemic control after metformin monotherapy [24]. Other studies have examined the role of rs72552763 (M420del) [7,19,25,26], rs12208357 (R61C) [19,25,26], rs34130495 (G401S) [21], rs34059508 (G465) [25], or rs2282143 (P341L) on glycemic control [27]. However, some of them have been mostly associated with the gastrointestinal side effects of metformin due to the decreased activity of the *OCT1* transporter [28,29,30].

The impact of genetic variants in *SLC22A1* on glucose control and the metformin response could have a major impact on diabetes treatment targets. Given the diversity of populations and the limited pharmacogenetics studies in Mexico, along with variable outcomes, this study aims to determine the genotype and allele frequencies of seven *SLC22A1* genetic variants that had been reported in Hispanic and Caucasian populations (rs622342, rs628031, rs72552763, rs12208357, rs34130495, rs34059508, and rs2282143) and to assess their association with glycemic control in individuals with T2DM undergoing metformin monotherapy in Northern Mexico.

## 2. Materials and Methods

### 2.1. Study Design and Population

A cross-sectional study was carried out, including 110 patients who were previously diagnosed with T2DM from Chihuahua (North State in Mexico) and attending public health institutions. Data were obtained from June 2019 to July 2021. The inclusion criteria were age ≥18 years, T2DM clinical diagnosis, with metformin monotherapy for at least six months, with at least one HbA1c% measurement in their clinical records; we did not have control of pharmaceutical technology aspects such as formulation, production, administration, and prescription. Patients with cancer and pregnant or nursing women were excluded. Medical history, demographic, anthropometric, and biochemical data were obtained from clinical records. Participants were classified into two groups according to their HbA1c levels: an adequate glycemic control group (<7.0% HbA1c) and inadequate glycemic control group (≥7.0% HbA1c) [2]. This study was approved by the Institutional Research Ethics Committees (CI-020-19 and R-2020-805-028) and it was conducted in accordance with the Good Clinical Practice guidelines and the and the Declaration of Helsinki [31]. All participants provided informed consent.

### 2.2. SCLC22A1 Genotyping

Genomic DNA was extracted from peripheral blood leucocytes by using a Masterpure Epicentre Kit (Thermo Scientific, Madison, WI, USA) according to the manufacturer’s manual. *SLC22A1* genotyping was performed using real-time PCR on the QuantStudio 3 system. The process utilized commercial TaqMan^®^ probes and SNP genotyping assays (Applied Biosystems, Foster City, CA, USA) to analyze the following polymorphisms: rs622342 (assay ID: C_928527_20), rs628031 (Met408Val, assay ID: C_8709275_60), and rs72552763 (Met420del, assay ID: C_34211613_10). The polymorphisms rs12208357 (R61C), rs34130495 (G401S), rs34059508 (G465R), and rs2282143 (P341L) were genotyped by polymerase chain reaction-restriction fragment length polymorphism (PCR-RFLP) based on Yang et al.’s method [27]. Briefly, the PCR reactions were conducted in 25 µL total volume using 80 ng of genomic DNA, 0.1 µM of each primer, and 1X Go *Taq* Master Mix (Promega Corporation, Madison, WI, USA) on the Agilent SureCycler 8800 (Santa Clara, CA, USA). The PCR conditions included an initial denaturation at 94 °C for 2 min, followed by 35 cycles (denaturation at 94 °C for 50 s, annealing at 58 °C for 50 s, and extension at 72 °C for 5 min), and a final extension at 72 °C for 10 min. The PCR products were digested with *HhaI*, *ApeKi*, and *BsaXI* restriction enzymes (New England BioLabs, Ipswich, MA, USA) for rs12208357, rs2282143, and rs34059508, respectively, and with the Cfr13I restriction enzyme (Thermo Scientific, Madison, WI, USA) for rs34130495, according to the manufacturer’s instructions. The products were visualized in a 3.0% agarose gel stained with ethidium bromide. Primers, restriction enzymes, and fragments are presented in Supplementary Table S1. Randomly, 20% of the qPCR and PCR-RFLP samples were repeated to confirm the results. We used internal controls in each PCR and the results were consistent.

### 2.3. Statistical Analysis

The mean ± standard deviation (SD) or median and interquartile range (IQR, Q1–Q3) were used to summarize normally or non-normally distributed quantitative data, respectively. Demographic characteristics and biochemical measurements between the two groups were compared by student’s *t*-test or the Wilcoxon rank sum (Mann–Whitney U) test. Pearson’s chi-square (*Χ*^2^) test was used to compare categorical variables and the Hardy–Weinberg equilibrium (HWE) test was used to estimate the number of homozygous and heterozygous variant carriers based on their allele frequencies. Quantitative measures were compared among genotypes by ANOVA or Kruskal–Wallis tests, followed by Bonferroni or Dunn’s tests. Logistic regression analysis was used to determine the association between the *OCT1* genotype and glycemic control (<7.0% HbA1c vs. ≥7.0% HbA1c) by inheritance models: the codominant model compares the three genotypes MM, Mm, and mm; the dominant model compares MM vs. Mm + mm, recessive Mm + MM vs. mm; and the over-dominant model compares MM + mm vs. Mm (MM, major allele genotype; Mm, heterozygous genotype; and mm, minor allele genotype, considering M as the major allele and m as the minor). SNPs with minor allele frequency (MAF) ≤1% were excluded from the analysis.

The normal distribution of the HbA1c levels was not reached by transforming options (e.g., log, square, cubic). Therefore, we used quantile regression to relate HbA1c as a continuous variable and *SLC22A1* inheritance models, adjusting for potential confounders (sex, age, BMI, age at the time of diagnosis, disease evolution, and metformin dose). Only those variables that remained significant were included in the final models. Two diplotypes in *SLC22A1* (rs622342 and rs628031) were evaluated in the dominant model, including statistically significant covariates and interactions. We also included a model to test the interaction between these diplotypes and the metformin dose. The statistical analysis was performed with STATA software version 14.2 (Stata Corp LLC, College Station, TX, USA).

## 3. Results

### 3.1. General Characteristics of T2DM Patients

Out of 154 patients, 110 met the selection criteria. The study population included 67% women (*n* = 74) and 33% men (*n* = 36); among patients, 60% had obesity (BMI ≥ 30 kg/m^2^) and 70% showed adequate glycemic control (<7% HbA1c). Patients with poor glycemic control were younger and diagnosed with T2DM at a younger age; they showed higher glucose and HbA1c levels and lower HDL-c levels (Table 1) than those with adequate glycemic control. There were no statistically significant differences in other clinical and biochemical variables between the study groups. All patients were on metformin monotherapy, with 74.55% taking the immediate-release type and 25.45% taking the extended-release type (XR-metformin). The metformin dose ranged from 500 to 2550 mg/day.

### 3.2. Genotype and Allele Frequencies of SLC22A1 Gene Variants

*SLC22A1* gene variants were in HWE, except for rs622342 (*p* = 0.01). It should be noted that this variant was in HWE (*p* = 0.31) in the initial sample of 154 individuals, prior to excluding those without HbA1c data. No minor allele homozygous subjects were found for rs34130495, rs34059508, rs12208357, and rs2282143, and the heterozygous frequency was also low; therefore, no further analysis was conducted for these polymorphisms (Table 2).

### 3.3. Glycated Hemoglobin, Glycemic Control, and SLC22A1 Gene Variants by Inheritance Model

The HbA1c levels showed statistical differences in patients with the rs622342 and rs628031 variants in most of the inheritance models (Table 3). Higher HbA1c levels were found in individuals with the AA (codominant and dominant model) and AA + AC genotype (recessive model) of the rs622342, and in those with GG and AA or GG + AA genotypes (vs. GA in the codominant and over-dominant models) for rs628031. In the over-dominant model for rs628031, a significantly higher proportion of T2DM patients with either GG or AA genotypes had inadequate glycemic control (78.80%) compared to controls (58.40%) [*p* = 0.041]. The rs72552763 did not show significant differences in glycemic control in any inheritance models.

The association between glycemic control (HbA1c < 7% vs. HbA1c ≥ 7%) with the rs622342 and rs628031 variants was assessed by logistic regression. In the over-dominant model, we identified that rs628031 (GG + AA) homozygous T2DM patients had 2.7 (i.e., 1/0.37) higher odds of poor glycemic control (HbA1c ≥ 7%) than heterozygous patients. This association remained significant after adjusting for age (Table 4).

### 3.4. Multivariate Analysis of rs628031 and rs622342 with HbA1c Levels

A quantile regression analysis was conducted to assess the rs628031 and rs622342 diplotypes using a dominant model, where carriers of at least a minor allele in either of these two variants (rs628031-GG + rs622342 AC+CC, rs628031GA+AA + rs622342-AA, and rs628031-GA+AA + rs622342 AC+CC) were compared to homozygous subjects for the major allele in both (rs628031-GG + rs622342-AA), adjusting for potential confounders. Table 5 presents the four diplotype combinations.

Interaction effects between these variants and BMI were identified (model 1). Individuals carrying at least one minor allele of A-rs628031 exhibited reduced levels of HbA1c compared to individuals homozygous for both variants, considering the BMI. Similarly, although all patients received metformin therapy, the type and dose varied. Metformin type (*p* = 0.554) and dose (*p* = 0.485) were not independently associated with HbA1c levels. However, an interaction effect was observed for diplotypes and metformin dose (model 2). In this case, diplotypes rs628031-GG + rs622342 AC+CC and rs628031GA+AA + rs622342-AA had lower HbA1c levels than individuals homozygous for both variants, considering the effect of metformin dose.

## 4. Discussion

This study aimed to determine the genotype and allele frequencies of seven *SLC22S1* gene variants and evaluate their relationship with glycemic control and HbA1c levels in T2DM patients from Northern Mexico receiving metformin monotherapy. The results showed that rs622342 and rs628031 were significantly related to HbA1c levels and interactions between rs622342 and rs628031 diplotypes with BMI and metformin dose were observed.

Minor allele frequencies (MAF) of *SLC22A1* SNPs vary across populations. The frequency of the C-rs622342 allele has been reported from 23% to 54%, and in this study it was 32%, consistent with previous findings in Mexican and Caucasian populations [7,8,22,24,25,32]. Similarly, the frequency of A-rs628031 in our study was 25%, within the range of previous reports (13–44%) [6,8,21,22,29]. The frequency of the deletion allele in rs72552763 was similar to previous reports from Mexico [25], Argentina [27], and Scotland [26], and lower than other studies, in which the frequency of the “del” allele was nearly 100% [19,33]. The MAFs of the remaining *SLC22A1* gene variants (rs2282143, rs12208357, rs34059508, and rs34130495) were below 10%, consistent with reports in Caucasian, Latin American, and Mexican populations. However, in this study, SNPs’ allelic frequencies showed slight variations compared to those reported for Mexican ancestry in a Los Angeles (LA) population from the 1000 Genome Project (https://www.ensembl.org, accessed on 10 January 2025) [34]. Differences may be influenced by genetic backgrounds [13] (Table S2).

Deviations from HWE may be due to other genetic factors such as a protective allele, population admixture/stratification, inbreeding, deletions, or due to genotyping errors [35]. The observed HWE deviation inrs622342 may be due to the reduced study sample size after exclusion criteria (those lacking HbA1c% data). The allele frequencies were proportionally reduced and the observed number of heterozygous subjects increased; moreover, the selection was a non-random sample from the general population. Similar deviations from HWE have been reported in the same *SLC22A1* gene variants [24] and in other SNPs, such as trs72552763 [25]. Furthermore, the HWE deviation might suggest that the specific genetic variant and the disease phenotype are connected.

The association of the A allele of rs622342 with clinical outcomes in T2DM has yielded inconsistent results. The response to metformin in Mexicans, Dutch Caucasians, South Indians, and other populations with T2DM improves, as suggested by lower HbA1c levels [8,16,22,23]. Conversely, another study conducted in Mexican Mestizo patients receiving combination therapy with metformin found that this polymorphism did not show a significant impact on their glycemic control [7]. In accordance with these findings, in our current study, A-rs622342 was not associated with glycemic control, defined by HbA1c levels below 7%. However, we observed that carriers of the C minor allele exhibited lower HbA1c levels among T2DM patients from Northern Mexico.

The mechanism by which the intronic rs622342 variant may affect *OCT1* transporter function remains poorly understood. Intronic variants can impact mRNA stability, alter gene splicing, or influence regulatory elements [36], which may contribute to variations in *OCT1* function and, consequently, metformin efficacy. In contrast, the rs622342 variant, associated with the C allele, has been linked to defective *OCT1* transporter function in a clinical study involving T2DM patients receiving combined therapy with metformin and glimepiride. This variant could impair hepatic metformin uptake, reducing its therapeutic effect [37]. These findings highlight that genetic variability within the *SLC22A1* gene, particularly the rs622342 variant, may contribute to the suboptimal glycemic response to metformin in specific populations, including our study.

rs628031 is one of the most extensively studied *SLC22A1* SNPs, since it has been associated with a response to metformin [16]. In vitro expression studies have shown that *OCT1* 408Met>Val does not change its transport ability [38]. Furthermore, site-directed mutagenesis in the 408Met>Val transmembrane domain did not lead to functional changes [39]. Moreover, studies in human liver samples carriers -43T/T (intron 1) showed no significant difference in *OCT1* mRNA expression among genotypes of rs628031 [40]. A recent conformational study determined the functional relation of genetic human h*OCT1* variants, including rs628031, in which the minor allele reduced the transport activity by less than 10% [41]. In the same line, no significant impact of rs628031 on the metformin response has been observed in Caucasian, Mexican, and Iranian populations [20,21,22]. In contrast, HbA1c was significantly lower in Han Chinese populations with the homozygous genotype AA [19]. In our study, under an over-dominant model, patients with the rs628031-GA heterozygous genotype had slightly lower HbA1c levels, whereas a study in a different Mexican population reported that the GA-genotype carriers showed a marginally lower increase in HbA1c levels than those with the GG genotype after a one-year follow-up [8]. Despite these studies, the mechanisms by which gene variants are related to drug pharmacokinetics and the ethnic variations in the loss of *OCT1* activity [13] remain unclear.

While a specific polymorphism may theoretically affect glycemic control in T2DM, the outcomes depend on a complex interplay of other genetic factors, gene–gene interactions, environmental factors, and biological redundancy. Becker et al. were the first to report an interaction of rs622342 with a multidrug and toxin extrusion (MATE) polymorphism, another transporter related to metformin excretion. Caucasian patients with the CC-rs622342 and the AA-rs2289669 genotypes had lower HbA1c % values [42]. While Marta et al. evaluated the rs622342 and rs72552763 interaction in the Mestizo Mexican population, observing that the haplotype combination of A-rs62234 and GAT-72552763 increased the risk of poor glycemic control by more than three times (OR = 3.067, *p* = 0.024) in T2DM patients receiving metformin treatment [25]. Ortega-Ayala et al. reported no significant association with the same diplotype; however, their study was performed in patients who received a combined therapy with metformin [7]. We analyzed a diplotype combination with rs622342 and rs628031. To the best of our knowledge, this is the first study to describe an interaction between these SNPs in patients from Northern Mexico. Interestingly, we observed a diplotype interaction with BMI and metformin dose related to HbA1c % levels. At the same dose of metformin, patients with rs628021-GG + rs622342-AC+CC or rs628031-GA+AA + rs622342-AA diplotypes showed lower levels of HbA1c than those with both homozygous major alleles.

Studying SNPs provides insight into how a single genetic variant influences T2DM glycemic control. Nonetheless, the diplotype association analysis offers a more comprehensive approach to understand how multiple genetic variants within a haplotype interact with each other and contribute to disease [43]. Moreover, using genomic databases (e.g., www.internationalgenome.org, accessed on 10 January 2025) and alternative analyses of population genetics for SNPs associated with multifactorial phenotypes can complement association studies by identifying trends across populations, thereby enhancing our understanding of heritability in health [44].

On the other hand, the translational implications of these findings are still under investigation. The Pharmacogenomics Knowledgebase (PharmGKB) summarizes how genetic differences impact medication response. Even though level 3 clinical annotations indicate low evidence supporting the association, some of the *SLC22A1* gene variants that we study (rs628031, rs622342, rs12208357, rs72552763, and rs2282143) are included, and data are still inconclusive to suggest their role in the metformin responses [45]. Furthermore, the Clinical Pharmacogenetics Implementation Consortium (CPIC) [46] and the Food and Drug Administration (FDA) guidelines [47] do not have information on metformin pharmacogenetic clinical recommendations.

This study has several limitations. First, the small sample size and the low frequency of the minor allele homozygous genotypes constrained the statistical power for the analysis of some *SLC22A1* variants. The sample size also limited the logistic and multivariate regression analyses, although it did allow for the detection of significant differences in diplotype interactions with BMI and metformin dosage. Second, the cross-sectional design of the study restricted the evaluation of the metformin response and long-term patient outcomes, as only one HbA1c measurement was analyzed. Nevertheless, patients on metformin monotherapy for at least six months were included, and the time of T2DM onset and other individual characteristics were considered as potential confounders. Despite these limitations, this study provides valuable insights, especially in detecting genotype–genotype interactions. However, considering these limitations, the results should be taken with caution.

## 5. Conclusions

The rs622342 and rs628031 variants of the *SLC22A1* gene may influence HbA1c levels in patients with T2DM from Northern Mexico. Diplotypes of these gene variants interact with BMI or metformin doses, contributing to changes in patients’ HbA1c levels. These results require confirmation in cohort studies. Further research on the mechanisms and clinical relevance of these genetic variants for clinical decision making and personalized T2DM management is warranted. The goal is to enhance therapeutic outcomes and minimize adverse effects through tailored treatment approaches.

## Figures and Tables

**Table 1:** Clinical and biochemical markers in T2DM patients in total population and according to glycemic control.

Variable	Total Population Mean ± SD/Md (IQR) *n* = 110	Adequate Glycemic Control HbA1c < 7.0% Mean ± SD/Md (IQR) *n* = 77 (70%)	Inadequate Glycemic Control HbA1c ≥ 7.0% Mean ± SD/Md (IQR) *n* = 33 (30%)	*p*
Male/Female	36/74	23/54	13/20	0.329
Age (years)	53.5 ± 9.52	54.7 ± 9.7	50.7 ± 8.7	0.045
Age at the time of diagnosis (years)	49.0 ± 8.9	50.2 ± 9.2	46.1 ± 7.5	0.032
Disease evolution (years)	3.0 (1.0–6.0)	3.0 (1.0–5.0)	3.0 (1.0–7.5)	0.905
Metformin dose (mg/day)	1500 (850–1700)	1700 (850–1700)	1500 (850–1700)	0.819
BMI (kg/m^2^)	31.4 (28.1–34.8)	30.8 (27.7–34.6)	32.0 (28.6–36.1)	0.321
SBP (mmHg)	127.6 ± 12.0	127.7 ± 11.9	127.2 ± 12.4	0.854
DBP (mmHg)	78.80 ± 7.98	79.1 ± 8.3	78.1 ± 7.14	0.568
Glucose (mg/dL)	121 (100.0–149.0)	107.0 (97.0–124.0)	157.5 (144.0–213.5)	<0.001
HbA1c (%)	6.4 (5.9–7.4)	6.1 (5.8–6.4)	8.2 (7.4–9.5)	<0.001
Total cholesterol (mg/dL)	183.94 ± 39.95	184.4 ± 41.9	182.9 ± 35.9	0.872
Triglycerides (mg/dL)	166.0 (133.0–224.0)	167.5 (140.0–222.0)	161.0 (132.0–249.0)	0.916
HDL-c (mg/dL)	43.5 (35.5–52.0)	47.0 (40.0–53.0)	35.0 (29.0–41.0)	<0.001
LDL-c (mg/dL)	99.84 ± 38.70	99.4 ± 40.1	101.3 ± 35.3	0.862
VLDL-c (mg/dL)	33.2 (26.6–44.8)	33.5 (28.0–44.4)	32.2 (26.4–49.8)	0.916
Creatinine (mg/dL)	0.85 (0.7–1.0)	0.9 (0.7–1.1)	0.8 (0.7–0.9)	0.141

*Χ*^2^ test, Wilcoxon’s rank sum test (Mann–Whitney U), or Student’s *t*-test were used for nominal, non-normally, or normally distributed variables, respectively. IQR, interquartile range; SD, standard deviation; BMI, body mass index; SBP, systolic blood pressure; DBP, diastolic blood pressure; HbA1c, glycated hemoglobin; HDL-c, high-density lipoprotein cholesterol; LDL-c, low-density lipoprotein cholesterol; VLDL-c: very low-density lipoprotein cholesterol.

**Table 2:** Allele and genotype frequencies of *SLC22A1* polymorphisms in T2DM population.

SNP	Allele Frequency *n* (%)		Genotype Frequency *n* (%)			HWE *p*
rs622342	A	C	AA	AC	CC	0.01
	149 (68.0)	69 (32.0)	44 (40.0)	61 (56.0)	4 (4.0)	
rs628031	G	A	GG	GA	AA	0.85
	165 (75.0)	55 (25.0)	63 (57.0)	39 (36.0)	8 (7.0)	
rs72552763	GAT	Del	GAT/GAT	GAT/del	del/del	0.16
	171 (78.0)	49 (22.0)	63 (57.0)	45 (41.0)	1 (2.0)	
rs12208357	C	T	CC	CT	TT	0.90
	211 (96.0)	9 (4.0)	101 (92.0)	9 (8.0)	0 (0.0)	
rs2282143	C	T	CC	CT	TT	0.92
	212 (96.0)	8 (4.0)	102 (93.0)	8 (7.0)	0 (0.0)	
rs34059508	G	A	GG	GA	AA	1.0
	219 (99.0)	1 (1.0)	109 (99.0)	1 (1.0)	0 (0.0)	
rs34130495	G	A	GG	GA	AA	1.0
	219 (99.0)	1 (1.0)	109 (99.0)	1 (1.0)	0 (0.0)	

*p* > 0.05 for genotype frequency Hardy–Weinberg equilibrium (HWE), *Χ*^2^ test.

**Table 3:** HbA1c values and glycemic control by *SLC22A1* gene inheritance models.

Inheritance Model	Total HbA1c (%) Median (IQR)	*p*	Adequate Glycemic Control HbA1c < 7.0% *n* (%)	Inadequate Glycemic Control HbA1c ≥ 7.0% *n* (%)	*p* **
rs622342 Codominant					
AA	6.8 (6.1, 7.9) ^a^	0.017 *^+^*	27 (35.0)	17 (51.5)	
AC	6.3 (5.8, 7.0) ^b^		45 (60.0)	16 (48.5)	0.160
CC	5.9 (5.6, 6.0) ^c^		4 (5.0)	0 (0.0)	
Dominant					
AA	6.8 (6.1–7.9)	0.025 *	27 (35.5)	17 (51.5)	0.118
AC + CC	6.3 (5.8–6.8)		49 (64.5)	16 (48.5)	
Recessive					
AA + AC	6.4 (5.9–7.4)	0.035 *	72 (94.7)	33 (100.0)	0.179
CC	5.9 (5.5–6.0)		4 (5.3)	0 (0.0)	
Over-dominant		0.156 *			0.300
AA + CC	6.6 (6.0–7.6)		31 (41.0)	17 (51.5)	
AC	6.3 (5.8–7.0)		45 (59.0)	16 (48.5)	
rs628031 Codominant					
GG	6.6 (5.9–7.8) ^a^	0.031 *^+^*	41 (53.20)	22 (66.70)	0.084
GA	6.1 (5.8–6.4) ^b^		32 (41.60)	7 (21.20)	
AA	7.4 (6.0–8.6) ^a^		4 (5.20)	4 (12.10)	
Dominant					
GG	6.6 (5.9–7.8)	0.050 *	41 (53.20)	22 (66.70)	0.192
GA + AA	6.1 (5.8–6.9)		36 (46.80)	11 (33.30)	
Recessive					
GG + GA	6.4 (5.9–7.3)	0.305 *	73 (94.80)	29 (87.90)	0.200
AA	7.4 (6.0–8.6)		4 (5.20)	4 (12.10)	
Over-dominant					
GG + AA	6.6 (5.9–7.9)	0.009 *	45 (58.40)	26 (78.80)	0.041
GA	6.1 (5.8–6.4)		32 (41.60)	7 (21.20)	
rs72552763 Codominant					
GAT/GAT	6.4 (5.9, 7.4)	0.129 *^+^*	41 (53.0)	22 (67.0)	0.325
GAT/del	6.4 (5.8, 6.8)		34 (44.0)	11 (33.0)	
del/del	5.6 (5.3, 5.8)		2 (3.0)	0 (0.0)	
Dominant					
GAT/GAT	6.4 (5.9–7.4)	0.289 *	41 (53.0)	22 (67.0)	0.192
GAT/del + del/del	6.3 (5.8–6.8)		36 (47.0)	11 (33.0)	
Recessive					
GAT/GAT + GAT/del	6.4 (5.9–7.4)	0.061 *	75 (97.0)	33 (100.0)	0.350
Del/del	5.6 (5.3–5.8)		2 (3.0)	0 (0.0)	
Over-dominant					
GAT/GAT + del/del	6.4 (5.9–7.4)	0.577 *	43 (56.0)	22 (67.0)	0.290
GAT/del	6.4 (5.8–6.8)		34 (44.0)	11 (33.0)	

*p*^+^ by Kruskal–Wallis’ test. * *p* by Wilcoxon’s rank sum test. *p* ** for *Χ*^2^ test. Dunn tests ^a–c^ different letters indicate significant differences between genotypes. IQR, Interquartile range; HbA1c, glycated hemoglobin. Genotyping for one *SLC22A1* rs622342 sample was not determined.

**Table 4:** Association between *SLC22A1* gene variants and glycemic control by inheritance models, unadjusted and adjusted for age.

Gene Variants	Model	OR (_95%_CI)	*p*	_adj_OR (_95%_CI)	*p*
rs622342 *	Dominant				
	CC + AC versus AA	0.52 (0.23–1.20)	0.120	0.54 (0.23–1.26)	0.157
	Over-dominant				
	AC versus AA + CC	0.65 (0.28–1.47)	0.301	0.63 (0.27–1.46)	0.284
rs628031	Codominant				
	GG	1.0		1.0	
	GA	0.41 (0.15–1.07)	0.069	0.41 (0.15–1.10)	0.076
	AA	1.86 (0.42–8.18)	0.410	2.80 (0.58–13.44)	0.203
	Dominant				
	GA + AA versus GG	0.57 (0.24–1.33)	0.195	0.60 (0.25–1.43)	0.252
	Recessive				
	GG + GA versus AA	2.51 (0.59–10.7)	0.212	3.77 (0.80–17.75)	0.093
	Over-dominant				
	GA versus GG + AA	0.38 (0.15–0.98)	0.045	0.37 (0.14–0.96)	0.042

_95%_CI, 95% confidence interval; OR, Odds ratio; _adj_OR, Odds ratio adjusted for age. * Codominant or recessive models for rs622342 were not analyzed due to the low frequency of homozygous minor allele.

**Table 5:** Multivariate analysis of the relation between HbA1c and dominant diplotypes of rs628031 and rs622342 (*n* = 108).

Variables	β (_95%_CI)	*p*
Model 1		
rs628031-GG + rs622342-AA	Ref	
rs628031-GG + rs622342 AC+CC	0.91 (−1.91, 3.74)	0.524
rs628031GA+AA + rs622342-AA	3.87 (−0.17, 7.9)	0.060
rs628031-GA+AA + rs622342 AC+CC	3.24 (0.24, 6.24)	0.034
BMI (kg/m^2^)	0.12 (0.065, 0.173)	0.000
rs628021-GG + rs622342-AC+CC × BMI	−0.05 (−0.13, 0.037)	0.267
rs628031-GA+AA + rs622342-AA × BMI	−0.14 (−0.27, −0.009)	0.036
rs628031-GA+AA + rs622342-AC+CC × BMI	−0.13 (−0.218, −0.04)	0.004
Model 2		
rs628031-GG + rs622342-AA	Ref	
rs628031-GG + rs622342-AC+CC	1.32 (−0.21, 2.85)	0.090
rs628031-GA+AA + rs622342-AA	1.68 (−0.58, 3.94)	0.143
rs628031-GA+AA + rs622342-AC+CC	−0.15 (−1.68, 1.38)	0.846
Metformin dose (mg/day)	0.0009 (0.0002, 0.002)	0.016
rs628021-GG + rs622342-AC+CC × Met dose	−0.001 (−0.002, −0.0002)	0.015
rs628031-GA+AA + rs622342-AA × Met dose	−0.001 (−0.003, −9.22 × 10^−6^)	0.048
rs628031-GA+AA + rs622342-AC+CC × Met dose	−0.0003 (−0.001, 0.0007)	0.552

β, regression coefficient; _95%_CI, 95% confidence interval; Ref, Reference; BMI, body mass index, Met = metformin, pseudo R^2^ = 0.10.

## Data Availability

Data are publicly unavailable due to privacy and ethical restrictions. Further inquiries can be directed to the corresponding authors.

## Supplementary Materials

The following supporting information can be downloaded at: https://www.mdpi.com/article/10.3390/genes16020139/s1, Table S1: Primers and restriction enzyme used for *SLC22A1* genotyping. Table S2: Minor allele frequency of *SLC22A1* gene variants among T2DM population.
