# Supplementary material for: Genetic Variants of SLC22A1 rs628031 and rs622342 and Glycemic Control in T2DM Patients from Northern Mexico
Source: Genes (Basel). 2025 Jan 24;16(2):139. doi: 10.3390/genes16020139 (PMC11855146; doi:10.3390/genes16020139)
Supplement: Supplementary file 1 [file genes-16-00139-s001.zip › genes-3379827-supplementary.pdf]

Table S1. Primers and restriction enzyme used for *SLC22A1* genotyping.

SNP	Nucleotide change	Primer sequence<br>F: 5´-3´<br>R: 3´-5´	Size bp	Enzyme	RFLP pattern bp
**rs12208357**	C/T	F: TGATCAGATGGCCACGTGCATT<br>R: GAGCCGGGCGTGTCATACAC	607	*HhaI*	CC: 26, 83, 87, 100 y 311<br>CT: 26, 83, 87, 100, 170 y 311<br>TT: 26, 100, 170 y 311
**rs2282143**	C/T	F: TGCCCCTTGTCATGGGTGTGAAGC<br>R: AGCGTGCTGATTCTGCCTGGA	267	*ApeKI*	CC: 267<br>CT: 86,181 y 267<br>TT:86 y 181
**rs34130495**	G/A	F: CTCAGGTTCACGGACTCTGTGCT<br>R: CACTGTGCACGGCCCCTCAAT	381	*Sau96I*	GG: 14, 34, 110 y 223<br>GA: 14, 34, 110, 223 y 257<br>AA: 14, 110 y 257
**rs34059508**	G/A	F: TGTTGCCCTGTGCTGCAAATCTC<br>R: GCCCACTGCCGAGCTGCAAAA	870	*Bsa*XI	GG: 166, 346 y 358<br>GA: 166, 346, 358 y 524<br>AA: 346 y 524

F, forward primer; R, reverse primers; bp, base pairs.

Table S2. Minor allele frequency of *SCL22A1* gene variants among T2DM population.

Population	Minor allele frequency (MAF)							Ref
	rs622342 C	rs628031 A	rs72552763 del	rs2282143 T	rs12208357 T	rs34059508 A	rs34130495 A	
**This study**	0.32	0.25	0.22	0.04	0.04	0.01	0.01	
***Mexican ancestry LA**	0.41	0.11	0.37	0.02	0.02	0.04	0.01	[34]
**South Mexico**	0.37	0.18	-	-	-	-	-	[8]
**North Mexico**	-	0.15	0.38	-	0.02	-	-	[21]
**South Mexico**	0.36	-	0.24	-	0.02	0.004	-	[25]
**South Mexico**	0.41	-	0.35	-	-	-	-	[7]
**South Mexico**	-	0.13	-	-	-	-	-	[6]
**Argentine**	-	-	0.20	0.02	0.05	0.06	0.09	[27]
**Javanese-Indonesia**	-	-	0.98	-	-	-	-	[33]
**Han Chinese**	-	0.27	1	-	0.01	-	-	[19]
**Jordanian**	0.23	-	-	0.02	-	-	-	[24]
**Soth Indian**	0.54	-	-	-	-	-	-	[23]
**Caucasian**	0.37	0.440	-	-	-	-	-	[22]
**Scotland**	-	-	0.20	-	0.07	-	-	[26]

*The 1000 genome database samples are from healthy people.
